# The processing of body expressions during emotional scenes: the modulation role of attachment styles

**DOI:** 10.1038/srep44740

**Published:** 2017-03-17

**Authors:** Yuanxiao Ma, Xu Chen, Guangming Ran, Haijing Ma, Xing Zhang, Guangzeng Liu

**Affiliations:** 1 Faculty of Psychology, Southwest University (SWU), Chongqing 400715, China; 2 Research Center of Mental Health Education, Southwest University (SWU), Chongqing, 400715, China; 3 Institute of Education, China West Normal University, Nanchong, 637002, China; 4 School of Foreign Languages for Business, Southwestern University of Finance and Economics (SWUFE), Chengdu, 611130, China

## Abstract

There is broad evidence indicating that contextual information influence the processing of emotional stimuli. However, attachment theory suggests that attachment styles contribute to the ways in which people perceive emotional events. To shed light on whether the processing of body expressions during different emotional scenes is modulated by attachment styles, attachment-related electrophysiological differences were measured using event-related potentials. For avoidantly attached group, our results suggested that larger N170 amplitudes were educed by neutral bodies than angry bodies, which was found only in neutral scene. Moreover, significant differences were found in P300 amplitudes in response to angry bodies compared with neutral ones only during angry scene. However, securely and anxiously attached individuals were associated with larger P300 amplitudes in response to angry bodies versus neutral ones in both emotional scenes. The current study highlights the characteristics of cognitive processing of attachment styles on body expressions during different emotional scenes, with the variation of N170 and P300 amplitude in different emotional scenes as the best example.

Human beings are born with sociality with varies levels of social interactions. Different information occurs to us every day. In natural viewing conditions, different stimulus categories carrying affective information, such as facial expression, bodily expression, and backgrounds, may all be relevant and processed together, and these information streams may interact^1^. Thus, a fundamental element and prerequisite for these interactions is the ability to perceive and interpret the feelings of others and to align the own behavior in an adaptive manner^2^. Expression especially facial and bodily expressions are of eminent importance for social interaction as they convey information about the emotional state and are thus of great evolutionary relevance. According to the predominant “basic emotion” approach, the perception of emotion in faces or bodies is based on the rapid, automatic categorization of prototypical, universal expressions. Consequently, the recognition of facial and bodily expressions has traditionally been studied by using isolated, de-contextualized, static pictures of emotional stimulus that maximize the distinction between categories^3^,^4^,^5^. The scene information in which a single stimuli is embedded, as well as its influence on the processing of emotional content of a stimulus, are neglected. However, in everyday life, an individual’s expression is not perceived in isolation, but almost always appears within a situational context, which may arise from other people, the physical environment surrounding the face, as well as multichannel information from the sender^6^. Thus, the perception of facial expressions is presumably always influenced by contextual variables. There is accumulating evidence that visual scene play a crucial role of contribute to the way emotional stimuli are processed and interpreted by the brain^6^,^7^. For example, it has been shown that face processing is influenced by preceding contextual sentences^8^,^9^. Similarly, other studies have demonstrated that emotional face processing is modulated by gaze direction^10^,^11^, the posture of the body to which the face belongs^12^,^13^, or the surrounding visual scenery^14^,^15^.

Yet, how people respond to emotional stimuli may depend in part on individual differences in how they maintain social bonds, namely their attachment style^16^,^17^,^18^,^19^. Attachment theory provides a conceptual framework for understanding individual’s behavior and emotion regulation, as well as on how attachment-relevant stimuli are perceived and interpreted later in life^20^,^21^,^22^. According to Bowlby^21^, the internal working model of attachment is gradually internalized in the interaction between infants and their primary caregiver (usually the mother). The attachment system is activated when infants experience negative life events (e.g., maternal separation, hunger, illness, etc.), leading them to seek protection and comfort from attachment figures. However, when the need for proximity is not reliably met by supportive attachment figures, an insecure attachment style is developed^21^. Differences in attachment style may predict selective bias in attention toward certain types of emotional information from the external environment^18^,^23^,^24^. Securely attached individuals can manage their emotions properly and restore emotional balance, so they are able to moderate responses to emotional events^25^. Anxiously attached individuals have a strong need for closeness, worry about relationships, and fear being rejected. Consequently, such individuals are thought to ‘hyperactivate’ the attachment system and become highly sensitive and vigilant to potential threat information, and devote more cognitive resources to attachment-related material^26^,^27^, especially to threat stimuli. As opposed to attachment anxiety, avoidantly attached individuals are characterized by compulsive self-reliance and a preference for emotional distance from others, these individuals are associated with the preferential use of deactivating strategies to prevent others from perceiving their internal emotional state^10^.

Attachment styles of people are also assumed to affect the development and maturation of the brain and to have long-lasting effects on brain structures and brain function^28^. Indeed, attachment-related differences in brain function have been reported in several fMRI studies^17^,^29^,^30^, whereas the time-sensitivity of event-related potentials (ERPs) is able to show the temporal brain dynamics of emotional processing at fine temporal resolution that behavioral and fMRI studies do not have. N170 component is associated with face sensitivity and reflects the configural features processing of human faces^31^,^32^. A higher activation of the N170 component reflects a higher need for face discrimination resources^33^,^34^. However, recent studies suggested that the recognition of human body expression is also mainly based on configural encoding^13^,^35^. Besides, P300 component may be particularly useful as it thought to be a measure of motivated attention reflecting the amount of “motivational relevance” perceived in a stimulus^36^. Since top-down control mechanisms occur at later processing stage and reflect in late ERP responses (>300 ms) interpreted as correlates of attention allocation, arousal, control and/or awareness^37^,^38^. For example, the deactivating strategy of avoidantly attached group is a product of an effortful strategy that occurs rather late in the information processing sequence^39^. Thus, the majority of the present study focused on the N170 and P300 component. The N170 and P300 has been employed in previous studies of attachment. For N170 component, insecurely attached mothers showed a more pronounced negativity amplitudes compared to secure mothers, besides, securely attached mothers showed a stronger P300 to face stimuli than insecurely attached mothers^34^. Similarly, Leyh and colleagues^32^ also suggested the negative target stimulus evoked a larger N170 amplitudes in insecure mothers than secure mothers, also, insecurely attached mothers exhibited less P300 amplitudes for the targeting of infant faces than securely attached mothers, especially in the condition of frequent negative infant faces. But the above researches did not separate anxious and avoidant attachment from insecure attachment. Moreover, researchers proved that anxiously attached individuals elicits greater P300 or LPP amplitudes in response to negative pictures^40^,^41^. However, there are no such findings regarding attachment avoidance.

To date, relatively little research has examined how attachment style influences the brain perceives the emotional stimuli of a person when this is presented in a emotional scene. Given important individual differences in the functioning of emotional processing among attachment style, it is worth finding out whether the influence of emotional scenes on processing of emotional stimuli are effected when attachment style attended. Using event-related potentials (ERPs), it is possible to fill this gap in the literature by examining how attachment style especially attachment anxiety and avoidance affect different stages of emotional processing during different emotional scenes. Understanding what role that attachment style played in the dynamics processing of emotional stimuli in emotional scenes can help us to better understand the specific processing mechanism of attachment styles and can also highlight the close link between personal relationships and physiological processes.

In summary, the purpose of the present study was to test whether participants’ response in processing of body expressions in different valences of emotional scenes is affected by their attachment styles. Based on the findings of our previous study, our hypothesis is as follows: the emotion processing bias to angry bodies would be significantly different during neutral and angry face scene within each attachment group. More specifically, because of the avoidant processing is result from the effortful inhibition^18^, the efficacy of the deactivating strategy was attenuated when the mental resources needed to maintain thought suppression were taxed by a high cognitive load^42^. Therefore, we hypothesized that the deactivating strategies of avoidantly attached individuals would be ineffective in processing angry bodies during angry face scene, the N170 and P300 amplitudes evoked by angry bodies may significant increase than those of neutral bodies in angry face scene other than neutral face scene. Anxiously attached individuals preferences for the use of hyperactivating strategies^41^,^43^ and may invest more attentional resources on angry bodies. Thus, we predicted that greater N170 and P300 amplitude would be observed in response to angry bodies in both emotional scenes. An secure attachment style is associated with the ability to perceive people’s emotions, to decipher them correctly and to respond to them properly^25^. Therefore, we did not expect differences in N170 amplitudes between neutral and angry bodies in both emotional scenes. For P300, we hypothesized angry bodies would evoked larger P300 amplitudes than neutral ones in any emotional scene.

## Methods

### Participants

We recruited about 300 Chinese college students to complete the Experiences in Close Relationships inventory (ECR)^44^ prior to the experiment, which was used to assess the participants’ attachment styles. Based on Chavis and Kisley’s criteria^45^, 60 participants (31 women; mean age, 21.12 ± 1.58 years) met the criteria and were assigned to three attachment groups. Those scoring higher than 1 SD above the mean on the Anxiety subscale and lower than 1 SD below the mean on the Avoidance subscale were assigned to the anxiously attached group (n = 19, 10 women). Those scoring higher than 1 SD above the mean on the Avoidance subscale and lower than 1 SD below the mean on the Anxiety subscale were assigned to the avoidantly attached group (n = 21, 11 women). Those scoring lower than 1 SD below the mean on both of the Anxiety and Avoidance subscales were assigned to the securely attached group (n = 20; 10 women). All participants were healthy, right-handed, reported normal or corrected-to-normal vision, and had no history of neurological or psychiatric disorders. The study was approved by the local Southwest University ethics committee. In accordance with the approved guidelines, written informed consent was obtained from the participants prior to conducting pilot or formal experiments. All methods were carried out in accordance with the approved guidelines.

### Assessment of attachment style

The ECR questionnaire was designed as a dimensional measure of adult attachment styles, including anxiety and avoidance. The subscale (α = 0.94) includes an 18-item scale for attachment-related avoidance, which reflects avoidance of intimacy and interdependence, and an 18-item scale for attachment-related anxiety (α = 0.90), which reflects an individual’s concern about rejection and abandonment. Participants use a 7-point Likert scale to rate the extent to which they agree or disagree with each item, ranging from 1 (strongly disagree) to 7 (strongly agree). It is also a stable and test-retest reliable measure of an individual’s attachment style^46^. Alpha coefficients are generally near 0.90, and test-retest coefficients generally range from 0.50 to 0.75^43^.

### Materials

The priming and target stimuli employed by the present study were facial and body expressions respectively. In our social interactions, faces are not perceived in isolation and usually co-occur with a wide variety of visual, auditory, olfactory, somatosensory, and gustatory stimuli. Whole-body expressions are major visual stimuli that are naturally associated with faces. In fact, neuroscientific researches of the manner in which body expressions are processed have drawn increasing attention in recent times^1^,^47^. In addition, a recent study showed that body cues can better discriminate between intense positive and negative emotions and highlighted the role of the body in expressing and perceiving emotions^48^.

The emotional scene materials include 48 face photographs, which consisted of 24 female face photographs (12 angry and 12 neutral) and 24 male face photographs (12 angry and 12 neutral) were drawn from the Chinese Facial Affective Picture System database^49^. CFAPS is a database that has been normatively evaluated for affective valence and arousal. The face pictures were selected in such a way that they differed significantly in their valence (negative: 3.07 ± 0.44, neutral: 4.23 ± 0.52; *F* (1, 46) = 0.15; *p* < 0.001), but were similar in arousal (negative: 4.96 ± 0.97, neutral: 4.61 ± 0.29; *F* (1, 46) = 0.16; *p* = 0.101). Body stimuli were selected from the Bodily Expression Action Stimulus Test (BEAST)^50^. Before the formal ERP experiment, we conducted one pilot validation test using angry and neutral body as BEAST did not encompass pleasantness and arousal evaluation. The angry and neutral body expressions consisted of 100 grayscale images (64 angry, 36 neutral). The faces were blurred to avoid facial influence. Thirty-four subjects categorized the emotion expressed in the whole-body image by marking one out of two forced choices between angry and neutral. Subjects were also required to rate body expressions in terms of arousal and pleasantness using a 9-point scale for pleasantness: 1 (most unpleasant) to 9 (most pleasant); and arousal: 1 (least arousing) to 9 (most arousing). The validation data for the BEAST indicated that angry and neutral bodies were recognized well, with recognition accuracies of 90.92% and 88.56% respectively. We randomly selected 48 images of body expressions, which included 24 images of women (12 angry and 12 neutral) and 24 images of men (12 angry and 12 neutral). The angry and neutral body expressions differed significantly in their valence (negative: 3.08 ± 0.19, neutral: 4.74 ± 0.31; *F* (1, 46) = 2.64; *p* < 0.001) and arousal dimension (negative: 5.83 ± 0.29, neutral: 4.81 ± 0.25; *F* (1, 46) = 0.16; *p* < 0.001).

All images were identical in size, background, spatial frequency, contrast, and brightness. The viewing angle for each image was 2.8 × 3.7° and each image had a resolution of 72 pixels per inch. Each face and body expression was presented six times, resulting in a total of 288 stimuli (divided into three blocks of 96 trials each). The onset sequence of the four conditions was randomized across the trials.

### Procedure

The present study employed a modified cue-target paradigm in which the valence of the emotional scene (facial expression) was independent of the emotion of the subsequent body expression. The two factors of emotional scene (angry, neutral) and body expression (angry, neutral) were manipulated in the experiment, leading to four conditions.

**Figure 1:**
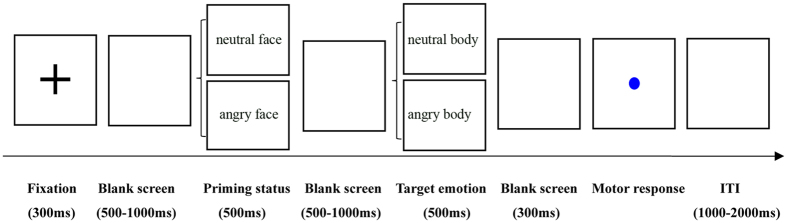
Schematic illustration of the experimental procedure.

Participants were seated in a quiet room approximately 90 cm from the computer screen and were instructed to try their best to avoid eye blinks and head movements. In each block, a trial was initiated by a 500-ms presentation of a black “+” followed by a white blank screen with an inter-stimulus interval that ranged from 500 to 1000 ms. Next, a emotional scene (either a neutral or angry facial expression) was shown for 500 ms. Participants only needed to passively view the emotional scene pictures. The emotional scene was then replaced with a variable 500–1000-ms blank screen, which was followed by the presentation of body stimuli for 500 ms. Participants were required to carefully observe the body stimuli and to evaluate the emotion of the body expression (fearful or neutral). However, they were not required to indicate their response. The body stimuli was then replaced by a 300-ms blank screen, which was followed by the presentation of a blue dot. Participants were required to press “F” when the blue dot followed a neutral body and to press “J” when the blue dot followed an angry body. The blue dot presentation was terminated by pressing a key. A schematic illustration of the procedure is shown in Fig. 1. Prior to the experiment, participants performed 12 practice trials to familiarize themselves with the experimental procedure.

### ERP recording

Electroencephalograms (EEGs) were recorded using a BrainAmps system (Brain Products, Munchen, Germany) from 64 scalp sites according to the 10–20 system positions with a reference at FCz^51^. A common average reference was recalculated. Vertical electrooculograms (EOGs) were recorded using electrodes placed below the right eye. Horizontal EOGs were recorded from the right orbital rim. Electrode impedance was maintained below 5 kΩ. Both EEG and EOG activities were amplified using a DC-100-Hz bandpass filter and continuously sampled at 500 Hz/channel for off-line analysis.

### ERP analysis

EEG activity was separately averaged for correct responses in each condition. The data were recomputed offline against an average mastoid reference. Epochs extended from −200 to 1000 ms relative to the onset of body expressions, using a 200-ms pre-stimulus baseline. Based on previous studies, the N170 component (130–190 ms) was determined over the Pz, POz, P1/2, P3/4, and PO3/4 electrodes^52^. The peak amplitude and latencies of N170 component were subjected to a repeated-measures analysis of variance (ANOVA) with attachment style as between-participants factor, and emotional scene, body expression, and location as within-participants factor. Given the absence of a sharply defined peak, the P300 component (300–500 ms) was observed and quantified as mean amplitudes at the Pz, POz, P1/2, P5/6, P7/8, and PO7/8 electrodes^53^. The mean amplitudes of P300 component were entered into a 3 × 2 × 2 × 3 repeated-measures ANOVA with emotional scene, body expression, and location as within-participants factor (right, midline, and left), and attachment style as between-participants factor. The ERP data were analyzed off-line using a BrainVision Analyzer (Brain Products).

### ICA cluster and source analysis

Using EEGLAB, we performed ICA cluster and source analysis^54^,^55^. EEG data were first decomposed in independent components (ICs) via independent component analysis (ICA). The ICs that had the same ERP morphology and scalp topography (spatial distribution) were clustered across participants. The IC scalp maps of each cluster were used for source dipole modeling. The Boundary Element Head Model was employed^56^.

## Results

### Behavioral results

**Figure 2:**
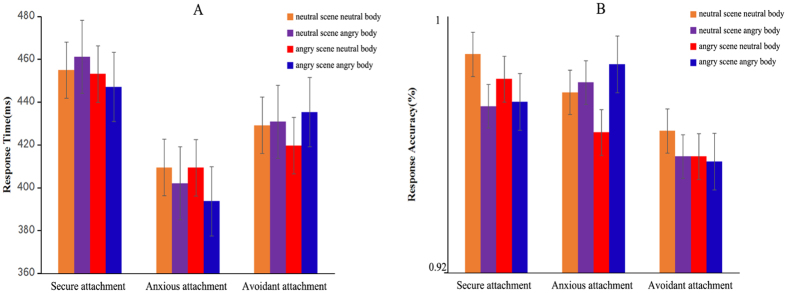
Behavioural responses on present task. Response time (**A**) and response accuracy (**B**) results for the body expressions in both emotional scenes within each attachment style. Data are represented as the mean and standard deviation.

Our results indicate that all participants had a greater than 80% accuracy rate for emotional bodies in both of emotional scenes. We excluded trials with error responses and reaction times (RT) above 1000 ms or below 200 ms in subsequent RT analyses. A repeated-measures ANOVA revealed a main effect of emotional scene, *F* (1, 57) = 4.15, *p* = 0.046, η^2^ = 0.068, indicating that participants responded faster to body expressions in angry scene (426.41 ± 9.43 ms) than neutral scene (431.30 ± 10.05 ms). However, there was no main effect of attachment style or body expression or any interaction. The corresponding repeated-measures ANOVA for accuracy rates did not yield any significant main effect or interaction effect (Fig. 2).

### ERP results

#### N170 effect

**Figure 3:**
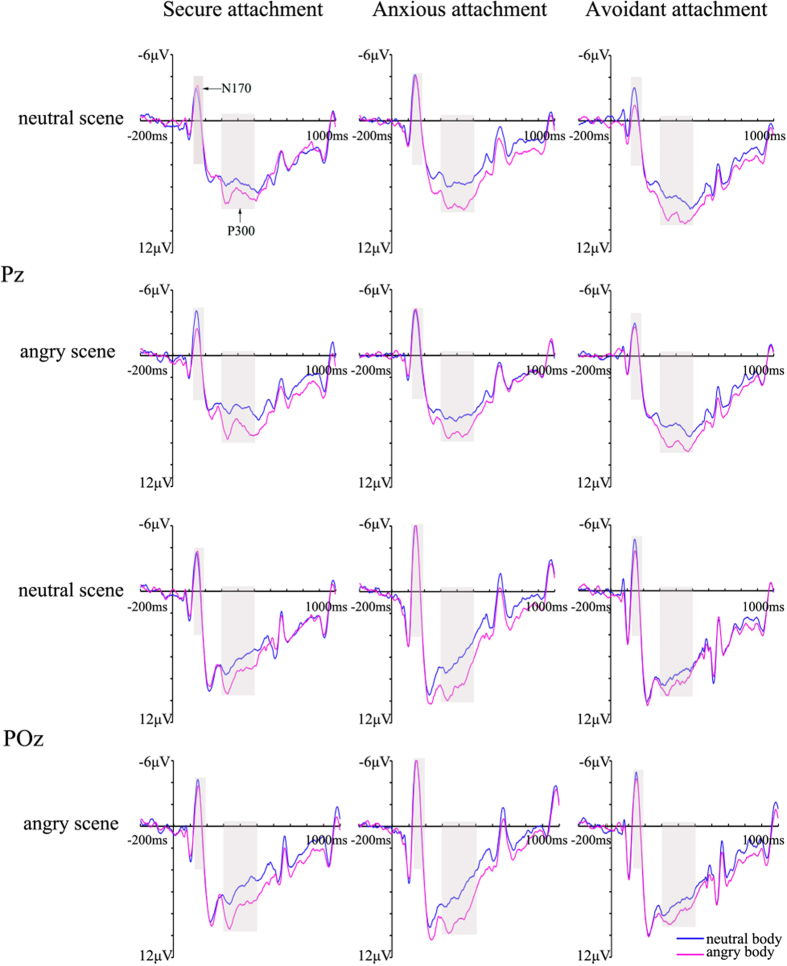
Grand average ERPs of the N170 and P300 component recorded at Pz and POz in response to neutral-body or angry-body during emotional scenes across attachment styles.

Significant attachment-related effects were not found for the N170 latencies and, therefore, are not reported here. The ANOVA of N170 peak amplitude revealed a significant main effect of emotional scene (*F* (1, 57) = 4.10, *p* = 0.048, η^2^ = 0.067), post-hoc ANOVAs revealed larger amplitudes for angry face scene were observed (neutral scene: −3.61 ± 0.49 μV, angry scene: −3.84 ± 0.47 μV). More importantly, we found a significant three-way interaction of emotional scene, body expression and attachment style (*F* (2, 57) = 5.37; *p* = 0.007, η^2^ = 0.16). For avoidantly attached group, neutral bodies (−3.26 ± 0.84 μV) induced a larger N170 amplitudes than angry bodies (−2.85 ± 0.84 μV, *p* = 0.034) in neutral scene, however, such difference were not found between neutral (−3.33 ± 0.78 μV) and angry (−3.36 ± 0.84 μV) bodies in angry scene. No significant N170 amplitudes difference were found between neutral and angry bodies in both emotional scenes among securely (NN (neutral bodies in neutral scene): 2.75 ± 0.86 μV; NA (neutral bodies in angry scene): 3.16 ± 0.86 μV; AN (angry bodies in neutral scene): 3.41 ± 0.80 μV; AA (angry bodies in angry scene): 2.91 ± 0.86) and anxiously (NN: 4.86 ± 0.88 μV; NA: 5.06 ± 0.88 μV; AN: 5.07 ± 0.82 μV; AA: 4.96 ± 0.89) attached groups (Fig. 3). No other significant main effect or interaction was observed.

#### P300 effect

For the P300 mean amplitudes, there was a significant main effect for emotional scene (*F* (1, 57) = 18.46, *p* < 0.001, η^2^ = 0.25), body expression (*F* (1, 57) = 23.94, *p* < 0.001, η^2^ = 0.30). Post-hoc ANOVAs revealed larger amplitudes for neutral scene was detected (neutral scene: 7.25 ± 0.42 μV; angry scene: 6.91 ± 0.41 μV), larger amplitudes yielded by angry bodies (7.67 ± 0.48 μV) versus neutral ones (6.49 ± 0.38 μV). Significant attachment-related two-way interactions were not found for the P300 and, therefore, are not reported here. More importantly, the four-way interaction between attachment style, emotional scene, body expression, and location reached significance, *F* (4, 114) = 2.86, *p* = 0.027, η^2^ = 0.09). Consistent with our hypothesis, subsequent analysis indicated that securely attached group is associated with larger P300 amplitudes over the left location electrodes in response to angry bodies compared with neutral bodies in both emotional scenes (NN: 6.49 ± 0.70 μV; NA: 7.54 ± 0.85 μV, *p* = 0.021; AN: 6.23 ± 0.67 μV; AA: 7.40 ± 0.82 μV, *p* = 0.009). Similarly, anxiously attached group exhibited enhanced P300 amplitudes to angry bodies compared with neutral bodies in both emotional scenes over the left location electrodes (NN: 5.95 ± 0.72 μV; NA: 7.55 ± 0.87 μV, *p* = 0.001; AN: 5.86 ± 0.69 μV; AA: 7.25 ± 0.84 μV, *p* = 0.003). Interestingly, with neutral scene, avoidantly attached group showed no difference in P300 amplitudes elicited by neutral and angry bodies over the left location electrodes (NN: 7.57 ± 0.69 μV; NA: 8.30 ± 0.83, *p* = 0.095), whereas P300 amplitudes evoked by angry bodies were larger than those evoked by neutral bodies with angry scene (AN: 6.90 ± 0.67 μV; AA: 8.01 ± 0.80 μV, *p* = 0.011) (Fig. 3).

### ICA clusters and estimated sources

**Figure 4:**
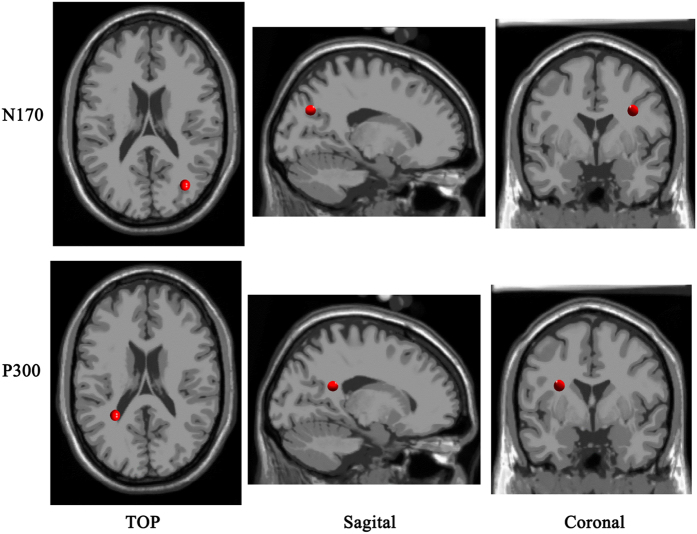
Results of the estimated sources for N170 and P300 component.

Independent component analysis (ICA) revealed four clusters of interest, namely the N170 and P300 clusters. The IC scalp maps of N170 cluster were characterized by temporooccipital distribution. The equivalent dipole source localization of ICs suggested that the N170 cluster had a source in right superior occipital gyrus (x = 32, y = −66, z = 31) with a RV of 7.08%. Moreover, we found that the topography associated with the ICs in the P300 cluster had a temporoparietal distribution, and the P300 cluster had a generator site in the parietal sub-gyral (x = −30, y = −44, z = 21) with a RV of 10.24% (Fig. 4).

## Discussion

The current study aimed to examine whether the dynamic processing of body expressions during the neutral- and angry-face scene is modulated by attachment styles. Accordingly, we identified ERP correlates of emotional processing bias by comparing ERPs elicited on trials with angry and neutral bodies during each emotional scene in individuals with different attachment styles.

Partly consistent with our hypothesis, brain responses to body expressions during each emotional scene differed as a function of attachment style. For anxiously attached group, angry bodies evoked greater P300 amplitudes compared with those evoked by neutral bodies during the both of emotional scenes in the left location electrodes. However, no difference was found in N170 amplitudes between neutral and angry bodies in both of emotional scenes, which is consistent with our hypotheses. This results can be understood easily. Previous research has claimed that the attentional system of anxiously attached individuals, who chronically rely on hyperactive strategies, tends to exaggerate their vigilance for threat-related stimuli in the environment, leading to an even more pronounced processing bias in favor of threat-related stimulation^22^. According to our results, this hyper-activation appears to involve mechanisms that work in the late stages (e.g., P300) of controlled attention allocation, but also at the early stages (e.g., N170) of stimulus analysis. The N170 is thought to be related to the structural encoding of the configural property, a larger N170 amplitude seems to be indicative of needing more processing capabilities^57^. Accordingly, the hyper-activation mechanism may involved the configural encoding of bodies as anxiously attached individuals activate more processing resources than other attachment groups, independent of emotional valence of the bodies (secure: −3.06 ± 0.82 μV; anxious: −4.99 ± 0.85 μV; avoidant: −3.13 ± 0.80 μV), even though did not reach statistical significance. As mentioned above, a heightened P300 amplitude reflects an increase in motivational engagement and mental resource allocation^58^,^59^,^60^. This suggests that anxiously attached individuals invested more attention resources in the processing of angry bodies regardless of neutral- or angry-face scene. Other ERP studies about attachment style that focused on late positive potentials found heightened P300 amplitudes in response to negative pictures in anxiously attached individuals, despite using completely different methodologies and stimuli^40^,^41^. This indicates that the use of hyperactive strategies for attachment anxiety depends on an individual’s internal state rather than on external emotional scenes. Thus, the effectiveness of hyperactive strategies adopted by anxiously attached group was consistent across completely different emotional scenes.

The hypothesis that securely attached group would allocate more motivational attention to the processing of angry bodies was supported by our findings. Just as our prediction, securely attached individuals displayed heightened P300 amplitudes in response to angry bodies compared neutral bodies regardless of emotional scenes, which means that these stimuli were processed more deeply. However, we are puzzled by the fact that similar results to body expressions between securely and anxiously attached group in both emotional scenes. Van Emmichoven and colleagues suggested that securely attached individuals lead to greater openness in the perception of positive and negative information^61^. Accordingly, securely attached individuals are capable of freely evaluating their emotions other than insecurely attached ones tend to either suppress or heighten the emotional experience in a regulatory effort. For example, a recent study, which examined emotional face processing in mothers using infant faces (with positive, negative, and neutral expressions) in an oddball paradigm, found that securely attached mothers had larger P300 amplitudes compared to insecurely attached mothers^34^. This may reflect a perceptual bias to social stimuli in securely attached group. Also, a recent study obtained a similar conclusion, they suggested secure attachment mothers were more sensitive to infants’ emotional expressions and needs (as reflected by larger P300 amplitudes)^32^. This fits well to theoretical assumptions and empirical findings from attachment theory, securely attached individuals seem to pay more attention to social stimuli which may enable them to detect social stimuli and to use them for social interactions. Besides, although no difference was found in N170 amplitudes between neutral and angry bodies during two emotional scenes in securely and anxiously attached group, N170 amplitudes evoked by anxiously attached group were larger than securely attached group even though did not reach statistical significance. So although securely attached individuals had similar results to those of anxiously attached individuals, we believe that it result from different internal processing mechanisms.

We discovered a very interesting result in the avoidantly attached group. A negative emotional processing bias only seen with angry scene but not neutral scene. This bias was reflected by (1) larger N170 amplitudes to neutral bodies than angry bodies in neutral scene while such difference was not found in angry scene; (2) enhanced P300 amplitudes to angry compared neutral bodies in angry scene over the left location electrodes while such difference was not observed in neutral scene. During neutral scene, the processing of angry bodies was inhibited or avoided because avoidantly attached individuals did not allocate more attentional resources to angry bodies than they did to neutral bodies. Indeed, avoidantly attached individuals have been characterized by a deactivation of the attachment behavioral system, leading to a down-regulation of interpersonally-experienced emotions^62^. Consequently, the deactivation strategy of attachment avoidance was available in neutral scene. From this perspective, it appears that avoidant processing bias results from the effortful inhibition of attachment-related material^18^,^42^.

Although some research suggests that the deactivation strategy can operate fairly effortlessly^63^,^64^, a contradictory processing bias was observed during angry-face scene as avoidantly attached individuals allocated more attentional resources to the processing of angry bodies versus neutral ones (for N170, there were no amplitude difference between neutral and angry bodies in angry scene, which means that the inhibition mechanism was out of work). Thus, the results of angry scene indicating the deactivation strategy of avoidantly attached individuals had failed. In other words, in the avoidantly attached group, the amount of cognitive resources used to suppress the processing of angry bodies was not sufficient during angry scene. Results obtained in angry scene support the idea that the efficacy of such strategies is attenuated when the mental resources needed to maintain thought suppression is taxed by a high cognitive load^42^. In angry scene, mental resources are not only needed to inhibit the perceptual processing of angry faces scene, but also to suppress angry bodies. In this case, there are reasons to believe that a lack of cognitive resources led to a weak inhibition of angry body processing. As a result, the deactivating strategy of attachment avoidance was ineffective, which was exemplified by the similar N170 and greater P300 amplitudes elicited by angry bodies compared neutral ones in angry-face scene.

The current study also confirmed that the deactivating strategies of attachment avoidance operated after the fact to suppress the processing of stimuli that had already been attended to or encoded. There are two main opinions regarding deactivating strategies for attachment avoidance. One view is that avoidantly attached individuals may be less attentive to attachment-related experiences^65^,^66^. Thus, these individuals’ defense mechanisms may operate preemptively to limit the amount of information that is encoded. Another opinion is that these individuals may reflect and elaborate less on the emotional information that they have encoded^42^. In this case, defense mechanisms may operate postemptively to suppress ideas and memories that have already been attended to or encoded^66^,^67^,^68^. The results of N170 and P300 indicates that deactivating strategies are actually after-event defense strategies. Furthermore, in the late ERP window, there was a cluster of interest, namely the P300 cluster. The P300 cluster had a dipole located in the temporoparietal region. Our results suggest that the elaborate processing of body expressions during different emotional scenes is based on the activation of neuronal populations in the left temporoparietal areas. For the generators of the P300 event-related potential, to the best of our knowledge, stimulus modality-specific contributions come from the inferior temporal and superior parietal cortex for visual stimuli^69^. The P300 performs an important set of cognitive processes, such as attention and working memory, and is dysfunctional in neurologic and mental disorders^70^. Thus, knowledge regarding the generators of P300 will be crucial for a better understanding of its cognitive significance and its continuing clinical application.

As far as we know, the present study is among the first ERP studies highlighting how emotional scenes effect on the processing of subsequent body expressions is modulated by attachment styles. Our results indicate that the emotion regulation strategy of attachment avoidance may be limited in processing continuous stimuli versus single stimuli. As mentioned above, the deactivating strategies employed by avoidantly attached individuals do not work in angry-face scene, which probably means that the effectiveness of this strategy may be impacted by the amount and valence of information to be processed, consistent with the view of Edelstein *et al*.^18^. Thus, the present study calls into question the traditional views that deactivating strategies are employed by avoidantly attached individuals in response to negative events.

## Additional Information

**How to cite this article:** Ma, Y. *et al*. The processing of body expressions during emotional scenes: the modulation role of attachment styles. *Sci. Rep.* **7**, 44740; doi: 10.1038/srep44740 (2017).

**Publisher's note:** Springer Nature remains neutral with regard to jurisdictional claims in published maps and institutional affiliations.
